# Elastofibroma Dorsi: An Uncommon Benign Pseudotumour

**DOI:** 10.1155/2008/756565

**Published:** 2008-03-12

**Authors:** C. R. Chandrasekar, R. J. Grimer, S. R. Carter, R. M. Tillman, A. Abudu, A. M. Davies, V. P. Sumathi

**Affiliations:** Paediatric Orthopaedic Surgery, The Royal Orthopaedic Hospital Oncology Service, Birmingham B31 2AP, UK

## Abstract

Elastofibroma dorsi is an uncommon benign soft tissue pseudotumour usually located at the lower pole of the scapula, deep to serratus anterior, and often attached to the periosteum of the ribs, presenting with long history of swelling and occasionally pain and discomfort. This lesion is usually seen in patients over the age of 50 years and is not uncommonly mistaken as a malignant tumour because of its size and location deep to the periscapular muscles. Review of the orthopaedic oncology database of 17 500 patients revealed that there were 15 patients with elastofibroma dorsi. There were 12 males and 3 females, mean age at diagnosis of 68.4 years range 51–79 years. The diagnosis was confirmed by MRI in 3 patients, excision biopsy in 3 patients, trucut biopsy in 8 patients and open biopsy in 1 patient. Eight patients had excision of the lesion which was symptomatic. There have been no recurrences. We highlight the clinical and radiological presentation of elastofibroma dorsi to increase awareness of its existence and management.

## 1. INTRODUCTION

Elastofibroma dorsi is an uncommon benign soft tissue pseudotumour usually located at the lower pole of the scapula, deep to serratus anterior, often attached to the periosteum of the ribs, presenting with a long history of swelling and occasionally pain and discomfort in elderly patients. Jarvi and Saxen [1] first described the condition in 1959 at the 12th Congress of Scandinavian Pathologists and subsequently published their work in 1961. They are slow-growing tumours in the region of inferior angle of the scapula. Clinical presentation is usually with swelling, discomfort, snapping of the scapula, and occasionally pain. Careful radiological assessment with MRI or CT can reveal bilateral lesions virtually assuring the diagnosis [2]. Because the pseudotumours are deep to the deep fascia and are often more than 5 centimetres in size, then there is a possibility of malignancy. If the lesion is unilateral, the MRI appearance of a poorly circumscribed, heterogeneous soft tissue mass, occasionally enhanced by gadolinium, makes it difficult to exclude a soft tissue sarcoma with complete confidence. This necessitates the need for biopsy to confirm the diagnosis. Often the presence of the swelling with symptoms, albeit mild, makes patients prefer surgical excision of the swelling. There are few case reports in the orthopaedic literature [3–6]. We describe our experience of treating 15 patients with elastofibroma dorsi.

## 2. MATERIALS AND METHODS

In a prospective database containing details of all the referrals to our orthopaedic oncology centre for over 20 years, we identified 15 patients with a diagnosis of elastofibroma dorsi (Table 1).

There were 12 males and 3 females. The mean age at diagnosis was 68.4 years (range 51–79 years). The mean duration of symptoms was 20 months (range 3–60 months). It was bilateral in 2 patients (13%). Swelling, discomfort, and occasionally pain were the presenting symptoms. In one patient, the swelling was first noticed by his wife. One patient was known to have symptomatic cervical spondylosis and previous surgery for cervical rib.

**Figure 1:**
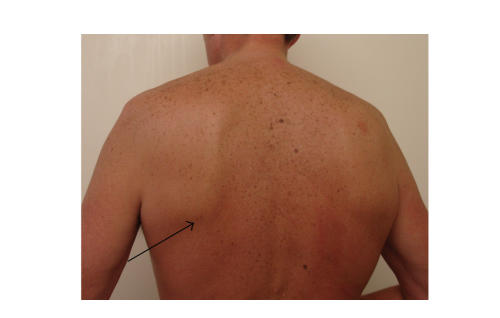
Arrow shows the typical location for elastofibroma dorsi.

Clinical examination showed a firm, deep, swelling in the infrascapular region (see, Figure 1) which was fixed to the rib cage. The swelling was not tender on palpation. The swelling was more prominent on forward flexion of the shoulder due to the inferior angle of the scapula moving forward.

Eight patients had a trucut biopsy, 1 patient had an open biopsy, 3 patients had excision biopsy, and 3 patients had their diagnosis based on clinical and MRI findings. Once the diagnosis was confirmed, 4 patients opted for nonoperative treatment with periodic assessment and 8 patients opted for excision of the swelling. During surgery, the swelling was found to be deep to lattismus dorsi and serratus anterior, and it was attached to the periosteum of the ribs in the infrascapular region. The aim of surgery was marginal excision of the swelling which was achieved in all patients. The wounds were closed with a drain in situ, and the drain was removed after 24–48 hours. The patients were followed for a mean period of 8.6 months (range 1–30 months). No patient had residual symptoms or local recurrence.

## 3. MR IMAGING FINDINGS

**Figure 2:**
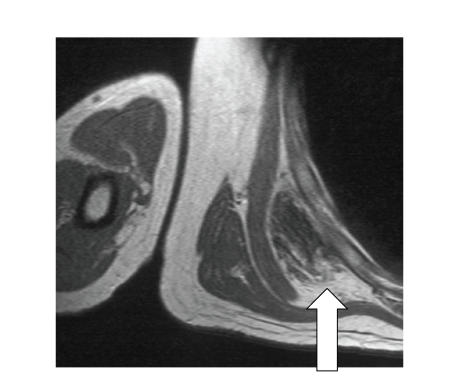
Axial T1-weighted MR image showing an elastofibroma as a soft tissue mass deep to the muscles and adjacent to the chest wall.

The lesion was anterior or caudal to the inferior pole of the scapula, and was deep in relation to lattismus dorsi, serratus anterior, and rhomboid muscles. It was a poorly circumscribed, unencapsulated soft tissue mass. Bilateral imaging and contrast enhancement was not routinely performed. The appearance of a soft tissue mass with signal intensity similar to skeletal muscle with regions of alternating high and low signal intensities on T1 and T2 weighted spin echo sequences in the typical subinfrascapular location was diagnostic of elastofibroma especially if the lesion was bilateral (see, Figure 2).

## 4. PATHOLOGY

**Figure 3:**
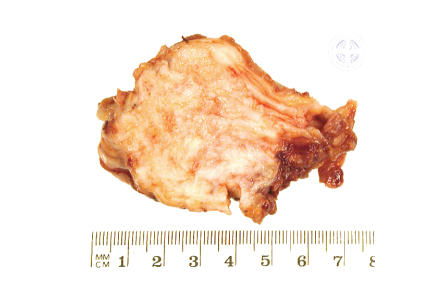
Macroscopic appearance of elastofibroma showing greyish white fibrous areas admixed with adipose tissue.

**Figure 4:**
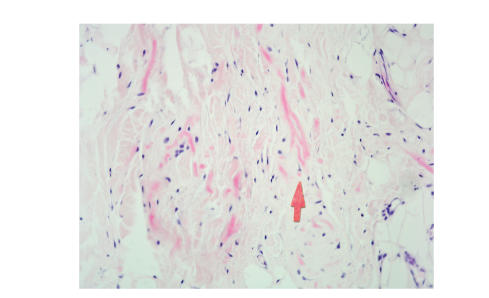
H&E stain showing branched and unbranched coarse elastin fibres admixed with collagen and mature adipose tissue (Arrow pointing at coarse branched elastin fibre).

**Figure 5:**
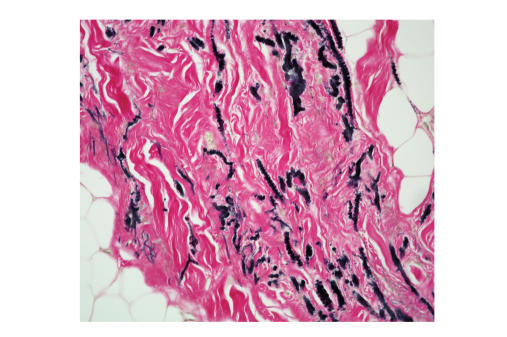
Weigert's elastin stain showing deeply staining branched and unbranched elastin fibres, between benign fibroblasts and adipose tissue.

Pathologists consider elastofibroma dorsi as a pseudotumour or tumour-like lesion. Macroscopically, the tumour was firm and ill-defined with a grey-white cut surface. The tumour varied in size from 3 to 10 cm. The average size was 7 cm. The tumour volume ranged from 14 to 367 cubic centimetres (average 92.5 cc). Histology showed that the tumour was hypocellular containing a mixture of benign fibroblasts, eosinophilic collagen, and elastin fibres. Elastin stain showed deeply staining branched and unbranched fibres exhibiting a central dense core and serrated margins. All specimens showed adipose tissue interspersed between the benign fibroblasts (see, Figures 3, 4, 5).

## 5. DISCUSSION

Elastofibroma dorsi is an uncommon benign lesion. Negamine et al. [7] have described a series of 170 patients from Okinawa. Genetic predisposition was reported with 32% of the 170 patients having a family history of elastofibroma. All the larger series of elastofibroma reported in the literature showed elastofibroma was commoner in females. In our series, it was commoner in males (80%).

Elastofibroma typically occurs in the subscapular or infrascapular region. It is also reported to occur in other sites like the axilla, ischial tuberosity, greater trochanter, posterior elbow, stomach, rectum, omentum, eye, hand [8], and foot. The site of occurrence was in the typical infrascapular region in our series.

Malghem et al. [9] in their review article on imaging study findings in elastofibroma dorsi noted the considerable disagreement about the need for obtaining a biopsy. In our series, the patients presented to the soft tissue sarcoma clinic. Trucut biopsy was performed at the time of consultation to obtain a definitive histological diagnosis. A series of 235 autopsies by Jarvi and Lansimies [10] found features of elastofibroma in the subscapular thoracic fascia in 29 of 119 (24%) females and 10 of 89 males (11%), all aged 58 or more. Giebel et al. [11] in a series of 100 autopsies found elastofibroma in 13 patients—10 males and 3 females. Naylor et al. [2] reported in their series of 12 patients that the tumour was bilateral in all the 9 patients in whom both sides of the chest was imaged and this indicated the benign nature of the swelling eliminating the need for biopsy.

Briccoli et al. [12] reported in their series of 9 patients that the tumour was bilateral in 3 patients (33%) and all of the 9 patients underwent surgical excision. Vastamaki [13] reported in a series of 5 patients that the diagnosis was clinical based on the presence of firm subscpular mass with long history. In our series, elastofibroma was unilateral in 13 patients (87%) and bilateral in 2 patients (13%). If there was a definitive radiological diagnosis with typical clinical presentation in asymptomatic patients, we deferred biopsy (3 patients).

Following clinical, radiological, and/or histological diagnosis, the patient was offered an informed choice: 11 of our patients opted for excision of the swelling and 4 patients opted for nonoperative treatment. Elastofibroma occurs after the 5th decade and the mean age in our series was 68.9 years consistent with other reported series. Large (> 5 cm) soft tissue swellings deep to the deep fascia strongly raise the possibility of a soft tissue sarcoma in this age group [14]. The average maximum dimension of the lesion was 7 cm. We had a low threshold to biopsy these lesions unless there was great confidence based on clinical and radiological grounds that the lesion was benign. This is reflected by the number of biopsies in our series.

The radiological and histological findings have been well described in various papers (Naylor et al. [2], Zembsch et al. [6], Malghem et al. [9], and Hayes et al. [15]).

Our series is the largest surgical series for this rare condition. The question of necessity for surgery for this benign lesion in an elderly population is legitimate. Informed choice should be offered to the patients, for various reasons surgery may or may not be chosen by the patient. If surgery was the preferred option, our series has shown that curative marginal resection can be performed safely in this age group. The periscapular region is highly vascular and the incidence of post operative haematoma should be borne in mind. There were no reported recurrences or other complications.

## 6. CONCLUSION

Elastofibroma dorsi is an uncommon benign soft tissue pseudotumour occurring in the infrascapular region of elderly patients. The size of the lesion, location deep to the deep fascia, and attachment to the ribs suggest the possibility of soft tissue sarcoma. Typical MRI findings especially if the tumour is bilateral confirm benign elastofibroma. If biopsy is performed to exclude soft tissue sarcoma, typical histological features are diagnostic of this benign lesion. Elastofibroma dorsi can be safely treated without surgery. If the patient chooses to have surgical excision, marginal excision of the lesion can be performed with minimal morbidity.

## Figures and Tables

**Table 1:** Details of 15 patients with elastofibroma dorsi.

Age at diagnosis	Sex	Side	Size (cms)	Duration of symptoms	Basis of diagnosis	Surgery	Follow up in months
71 yr	Male	R	6	6 months	Excision biopsy		2
77 yr	Male	R	7	6 months	Excision biopsy		1
51 yr	Male	R	5	2 months	Excision biopsy		1
65 yr	Male	L	10	60 months	Open biopsy	Excision	24
61 yr	Male	B	8.5	36 months	Trucut biopsy	Excision	5
70 yr	Female	R	10	12 months	Trucut biopsy	Excision	7
75 yr	Male	R	10	18 months	Trucut biopsy	Excision	10
68 yr	Male	L	9	6 months	Trucut biopsy	Excision	19
67 yr	Male	L	8	48 months	Trucut biopsy	Excision	2
76 yr	Male	L	10	3 months	Trucut biopsy	Excision	2
79 yr	Male	R	9	3 months	Trucut biopsy	Excision	2
66 yr	Female	R		36 months	Trucut biopsy		2
54 yr	Male	L	3	36 months	MRI		12
72 yr	Male	L	4.9	12 months	MRI		12
66 yr	Female	B	4	18 months	MRI		30

## References

[1] Jarvi OH, Saxen AE (1961). Elastofibroma dorsi. *Acta Pathologica et Microbiologica Scandinavica*.

[2] Naylor MF, Nascimento AG, Sherrick AD, McLeod RA (1996). Elastofibroma dorsi: radiologic findings in 12 patients. *American Journal of Roentgenology*.

[3] Hoffman JK, Klein MH, McInerney VK (1996). Bilateral elastofibroma: a case report and review of the literature. *Clinical Orthopaedics & Related Research*.

[4] Majo J, Gracia I, Doncel A, Valera M, Nunez A, Guix M (2001). Elastofibroma dorsi as a cause of shoulder pain or snapping scapula. *Clinical Orthopaedics & Related Research*.

[5] Nielsen T, Sneppen O, Myhre-Jensen O, Daugaard S, Nørbæk J (1996). Sub scapular elastofibroma: a reactive pseudo tumour. *Journal of Shoulder and Elbow Surgery*.

[6] Zembsch A, Schick S, Trattnig S, Walter J, Amann G, Ritschl P (1999). Elastofibroma dorsi: study of two cases and magnetic resonance imaging findings. *Clinical Orthopaedics & Related Research*.

[7] Nagamine N, Nohara Y, Ito E (1982). Elastofibroma in okinawa. A clinicopathologic study of 170 cases. *Cancer*.

[8] Kapff PD, Hocken DB, Simpson RHW (1987). Elastofibroma of the hand. *Journal of Bone & Joint Surgery*.

[9] Malghem J, Baudrez V, Lecouvet F, Lebon C, Maldague B, Vande Berg B (2004). Imaging study findings in elastofibroma dorsi. *Joint Bone Spine*.

[10] Jarvi OH, Lansimies PH (1975). Subclinical elastofibromas in the scapular region in an autopsy series. *Acta Pathologica et Microbiologica Scandinavica*.

[11] Giebel GD, Bierhoff E, Vogel J (1996). Elastofibroma and pre-elastofibroma—a biopsy and autopsy study. *European Journal of Surgical Oncology*.

[12] Briccoli A, Casadei R, Di Renzo M, Favale L, Bacchini P, Bertoni F (2000). Elastofibroma dorsi. *Surgery Today*.

[13] Vastamaki M (2001). Elastofibroma scapulae. *Clinical Orthopaedics & Related Research*.

[14] NICE (2005). Referral guidelines for suspected cancer.

[15] Hayes AJ, Alexander N, Clark MA, Thomas JM (2004). Elastofibroma: a rare soft tissue tumour with a pathognomonic anatomical location and clinical symptom. *European Journal of Surgical Oncology*.

